# Editorial

**Published:** 1869-01

**Authors:** 


### Editorial.

NOTHING NEW.—In the clinical reports in the *Medical Record* of New York, we find on page 463 the following:—

"CASE IV. *Renal Dropsy.—Treatment by the Bichloride of Mercury.*—In the case of a man with general dropsy of four months' standing, dependent on renal disease, Dr. Flint called attention to a *new* method of treatment by the use of the bichloride of mercury in small doses. On admission, his urine contained albumen and waxy casts. Corrosive sublimate was given, in doses of $\frac{1}{32}$ part of a grain with compound tincture of cinchona. The dropsy had now nearly disappeared."

What is here spoken of as a "*new method*" of treatment for renal dropsy, was proposed and practised in New York City more than twenty years since. It was adopted in the treatment of several cases in the New York Hospital, with reported benefit. We heard it spoken of in one of the College clinics, we think, by Prof. Willard Parker.

We were at that time residing in New York City, and kept a male patient, who had been disabled by general dropsy with highly albuminous urine for six months, on the following prescription during nearly eight months, with interruptions of only a few days at a time:—

℞. Tinct. Cinchona,--------------------- ℥iij.
Bichlorid. Hydrarg., ---------------- 1 gr.

Mix. Give one fluid drachm, in sweetened water, before each meal and at bedtime.

After he had been under treatment one month, the dropsical infiltrations had diminished so much that he could take considerable exercise, and the medicine was limited to three doses per day. At the end of eight months, there was still a little puffiness about the eyes in the morning, and in the feet and ankles at night. There were also slight traces of albumen in the urine. But he was able to do a moderate amount of manual labor every day. Ever since that time we have continued to use small doses of the bichloride of mercury in some cases of renal dropsy, with benefit. We have generally given it dissolved in tincture of cinchona bark. Given in this manner, and in doses of $\frac{1}{24}$ of a grain or less, we have seldom known it to affect the gums of the patient, even after several weeks' continuance.

---

Dr. Arthur E. Peticolas, Superintendent of the Eastern Lunatic Asylum at Williamsburg, committed suicide there on the morning of Nov. 28th, by leaping from a window of the building, and dashing out his brains. He was a distinguished physician, and formerly a professor in the medical college at Richmond. His mind had been unsettled for some time past.—*Medical and Surgical Reporter.*

---

It is proposed by the New York Medical College for Women to educate a body of professional nurses to attend freely or for a moderate charge, persons living in boarding-houses and like places, who are not able to secure regular attendance.—*Medical and Surgical Reporter.*

---

Money Receipts to December 23.—Drs. W. A. Gordon, $5; E. L. Holmes, 3; J. S. Sherman, 3; N. W. Abbott, 1; W. A. Knox, 3. J. H. Rauch, 9; M. Parker, 3; R. N. Isham, 6. M. O. Heydock, 6; J. P Ross, 3; W. C. Lyman, 3; John M. Woodworth, 2; A. Groesbeck, 3; J. W. Mill, .75; John Macalister, 6; J. M. Hutchinson, 3; M. F. DeWitt, 6; D. C. Roundy, 3; W. H. Byford, 3; G. C. Paoli, 5; J. N. McLane, 6; Latta & Sparklee, 6; A. Fisher, 6; Wm. Fleming, 3; D. B. Bobb, 3; J. B. Buchtel, 6; Hosme A. Johnson, 10; M. F. Bassett, 3; J. S. Hildreth, 6; R. C. Hamill, 6; Daniel Gard 1.50; J. C. Allaben, 3; W. W. Allport, 6; A. Hager, 3; D. H. Spickler, 6; S. S. Terry, 6; H. B. Wilcox, 5; D. E. Woodward, 6; John Conant, 6; G. Wheeler Jones, 3.25; E. Ballard, 6; D. V. Cole, 6; S. Wickersha[illegible], 3; C. H. Quinlan, 9; Wolcott & Markes, 6; Wm. J. Wheelan, 3; D. M. Creed, 7.50; Geo. Fredigke, 3; Henry Sweet, 6; H. A. Allen, 6; — Cleveland, 3; J. B. Walker, 3; Daniel Duckett, 6; Theron Nichols, 2.50; R. J. Patterson, 6; G. L. Henderson, 1.50; R. Ludlam, 6; Ira Hatch, 6; J. McLaughlin, 3; Edwin M. Park, 3.

**BELLEVUE PLACE,**

For the care and treatment of Nervous and Insane Invalids.

Address **R. J. PATTERSON, M. D.,**

Jan. 1, 1869. **Batavia, Ill.**

RELIABLE VACCINE MATTER CAN BE HAD OF
DR S. A. McWILLIAMS,
166 STATE STREET, CHICAGO.

### Mortality for the Month of November, 1868:—
### CAUSES OF DEATH.

| | |
|---|---|
| Accident, drowned | 6 |
| " fall, | 2 |
| " run over by wagon | 1 |
| " railroad, | 4 |
| Angina, | 2 |
| " scarlet fever & exhaustion, | 1 |
| Anæmia, | 1 |
| Apoplexy, | 1 |
| Aphthæ, | 1 |
| Births, premature, | 11 |
| " still, | 48 |
| " tedious | 2 |
| Bronchitis, | 5 |
| " and dropsy | 1 |
| Bowels, inflammation, | 2 |
| Brain, apoplexy of | 1 |
| " dropsy of, | 1 |
| " congestion of, | 6 |
| " " and convulsions | 1 |
| " " from injury, | 1 |
| " inflammation, | 1 |
| " softening of | 1 |
| Cancer, | 1 |
| " of breast, | 1 |
| Carbuncle and Convulsions, | 1 |
| Cholera infantum, | 2 |
| " morbus, | 1 |
| Convulsions, | 44 |
| " puerperal | 1 |
| " from overdose of drugs, | 1 |
| Croup, | 8 |
| " diptheretic, | 1 |
| " membranous | 1 |
| Cyanosis, | 1 |
| Debility | 3 |
| " general, | 4 |
| Delirium tremens, | 1 |
| Deficient vitality, | 1 |
| Diphtheria, | 10 |
| Diarrhœa, | 3 |
| " chronic, | 9 |
| Dropsy, | 5 |
| " peritonitis and gangrene, | 1 |
| Dysentery | 5 |
| " chronic | 3 |
| Epilepsy | 1 |
| Enteritis, chronic, | 1 |
| " and whooping-cough, | 1 |
| Erysipelas, | 6 |
| Fever, congestive, | 3 |
| " puerperal, | 2 |
| " remittent | 2 |
| " and enterocolitis, | 1 |
| " " pneumonia | 1 |
| " scarlet, | 23 |
| " " malignant, | 2 |
| " " and dropsy, | 2 |
| " " and convulsions, | 1 |
| " " and gangrene of tonsils, | 1 |
| " " and diphtheria, | |
| " " and nephritis, | 1 |
| " " pericarditis | 1 |
| " typhoid, | 20 |
| " " and meningitis, | 1 |
| Gastro-enteritis, | 1 |
| Gangrene, traumatic, | 1 |
| Glottis, spasms of, | 1 |
| Heart, dropsy of, | 1 |
| " disease of, | 5 |
| " " following parturition, | 1 |
| " valvulalar disease of, | 1 |
| " mitral valve, disease of, | 1 |
| Hernia strangulated | 1 |
| " incarcerated, | 1 |
| Hydrothorax & dropsy | 1 |
| Hydropericardium and paralysis, | 1 |
| Hip disease | 1 |
| Hydrocephalus, | 5 |
| " acute, | 1 |
| Injuries, | 1 |
| Inanition, | 8 |
| Intemperance and exposure, | 1 |
| Impaired nutrition, | 1 |
| Kidneys, Bright's disease of, | 3 |
| Laryngitis, | 2 |
| Liver, inflammation of | 1 |
| Lungs, congestion of | 3 |
| " " and pneumonia, | 1 |
| " effusion of, | 1 |
| " emphysema of, | 2 |
| " hemorrhage of, | 2 |
| " paralysis of, | 1 |
| " " and old age, | 1 |
| Measles, | 2 |
| Meningitis | 2 |
| " cerebro-spinal, | 3 |
| " tubercular, | 5 |
| Mitral, insufficiency of, and albuminuria, | 1 |
| Œdema pulmonium, | 2 |
| Old age, | 9 |
| " and fever intermittent, | 1 |
| Paratitis & fever scarlet, | 1 |
| Paralysis, | 1 |
| Peritonitis, | 5 |
| " acute, | 1 |
| " puerperal, | 1 |
| Pyæmia, | 1 |
| " and compound fracture of leg, | 1 |
| Phthisis pulmonalis, | 34 |
| Pneumonia, | 20 |
| " and convulsions | 1 |
| " typhoid, | 1 |
| Scrofula, | 1 |
| Stomach, cancer of, | 4 |
| Suicide, poisoning, | 1 |
| Tabes mesenterica, | 10 |
| Tongue, cancer of, | 1 |
| Teething, | 5 |
| " and scarlet fever, | 1 |
| Urethra, stricture of, | 1 |
| Uterus, cancer of, | 1 |
| " tumor of, | 1 |
| Whooping-cough, | 7 |
| " and convulsions, | 1 |
| Unknown | 5 |
| Total, | 401 |

COMPARISON.

Deaths in Nov., 1868, ..401 | Deaths in Nov., 1867, ..370 | Increase, .. 31
Deaths in Oct., 1868, ......... 448 | Decrease, .................... 47

AGES.

| | | | | | |
|---|---|---|---|---|---|
| Under 1 | 116 | 30 to 40 | 42 | 90 to 100 | 1 |
| 1 to 3 | 57 | 40 to 50 | 32 | 100 to 105 | 1 |
| 3 to 5 | 32 | 50 to 60 | 18 | Unknown | 2 |
| 5 to 10 | 34 | 60 to 70 | 10 | | |
| 10 to 20 | 15 | 70 to 80 | 10 | Total | 401 |
| 20 to 30 | 27 | 80 to 90 | 4 | | |

Males, ............221 | Females, ..........180 | Total, ............401

Single, ...........293 | Married, ......... 108 | Total, ............401

White, ............399 | Colored, ........... 2 | Total, ............401

NATIVITY.

| | | | | | |
|---|---|---|---|---|---|
| Foreign Chicago | 118 | England | 13 | Denmark, | 1 |
| Chicago | 73 | Germany | 45 | Sweden | 12 |
| Other parts U. S. | 61 | Holland | 3 | Unknown | 1 |
| Poland, | 1 | Ireland | 47 | | |
| Bohemia | 5 | Norway | 10 | Total, | 401 |
| Canada | 4 | Scotland | 4 | | |
| France | 2 | Italy, | 1 | | |

MORTALITY BY WARDS FOR THE MONTH.

| Ward. | Mortality. | Pop. in 1868. | One death in | Ward. | Mortality. | Pop. in 1868. | One death in |
|---|---|---|---|---|---|---|---|
| 1 | 8 | 9,094 | 1,137 | 14 | 27 | 14,839 | 549 |
| 2 | 14 | 13,074 | 934 | 15 | 33 | 21,078 | 639 |
| 3 | 26 | 15,076 | 580 | 16 | 18 | 15,465 | 859 |
| 4 | 16 | 17,796 | 1,112 | Bridewell, | 1 | | |
| 5 | 32 | 16,033 | 501 | County hosp. | 10 | | |
| 6 | 25 | 13,083 | 523 | Chi. River, | 4 | | |
| 7 | 42 | 25,492 | 607 | Mercy Hosp. | 4 | | |
| 8 | 33 | 15,813 | 479 | St. Luke's H's. | 1 | | |
| 9 | 18 | 19,297 | 1,072 | Immigrants | 10 | St. Jo. Orph. Asyl. | 1 |
| 10 | 13 | 12,925 | 994 | Hosp. of Alexian Bros., | 1 | Prot. Orph. Asylum | 1 |
| 11 | 14 | 14,340 | 1,024 | | | Hosp. for Women and Children, | 1 |
| 12 | 25 | 17,485 | 699 | | | Home for Friendless, | 2 |
| 13 | 21 | 11,164 | 531 | | | | |

Total, ................................ 401

A Clerical Surgeon.—Father Heylen, a catholic priest of Boom in Belgium, performed the Cæsarian operation on a young woman in order to baptize the infant before it died. The mother appears to have been living when the operation was commenced, but both mother and child succumbed. In his defence the priest said that he performed the operation in obedience to the direct instructions of the archbishop. These instructions are now to be cancelled, and the clerical surgeon tried for murder.—*Medical and Surgical Reporter.*

